# Clinical Researches on the Employment of the Tincture of the Flowers of the Colchicum in Articular Rheumatism, Simple or Gout, and in Neuralgia

**Published:** 1855-08

**Authors:** 


Clinical Researches on the Employment of the Tincture of the Flowers of the Colchicum in Articular Rheumatism, Simple or Gouty, and in Neuralgia. By Professor Forget. (Bull. Gén. de Thérap., 15 July, 1854, p. 1.)

Having been provided with some tincture of colchicum flowers, Prof. Forget administered it to eight patients suffering from the diseases mentioned, and puts forward his results in the following propositions, with that amount of reservation which the novelty of the subject and the small number of his observations demand.

1. The alcoholic tincture of colchicum flowers is a good remedy against acute articular rheumatism.

2. It is without any sensibly favorable operation in cases of chronic articular rheumatism, and in acute neuralgia.

3. Its physical, and probably its chemical, properties, its mode of administration, its physiological effects, and its therapeutical results, have considerable analogy with those of the tincture of the seeds of colchicum.

4. The efficacy of the tincture of colchicum flowers appears superior to that of the tincture of the seeds, in the treatment of acute articular rheumatism.

5. It should be administered in doses of ten to twenty drops and upwards three times a day.

6: Although it may act without producing gastric derangement I think that it is proper to increase the doses until several stools daily are produced; a point at which it is right to stop.—*Ibid*

